# Special Issue of *International Journal of Molecular Sciences* “Opioid Receptors and Endorphinergic Systems 2.0”

**DOI:** 10.3390/ijms22168365

**Published:** 2021-08-04

**Authors:** Carlo Ventura

**Affiliations:** 1 Istituto Nazionale di Biostrutture e Biosistemi (INBB), National Laboratory of Molecular Biology and Stem Cell Engineering—Eldor Lab, Innovation Accelerator, CNR, 40129 Bologna, Italy; carlo.ventura@unibo.it; 2 Department of Experimental, Diagnostic and Specialty Medicine (DIMES), School of Medicine, University of Bologna, 40138 Bologna, Italy

Opioid peptides exhibit a wide-ranging tissue distribution and control multiple tissue functions not only through reflex mechanisms involving the central nervous system or the modulation of neurotransmitter release, but also by acting directly at the cellular level by targeting selected receptor subtypes (μ, δ, and κ are among the most frequently expressed). Such a direct action has been shown to occur even in complex cell types, such as myocardial cells [1,2], which turned out to not only be the target but also the source for a number of biologically active end-products of endorphin genes, including the prodynorphin and the proenkephalin genes [3,4,5,6]. The binding of these peptides with their related receptors elicits paracrine and autocrine circuitries with profound implications in signal transduction, transcriptional dynamics, and stem cell fate [4,5]. Intriguingly, opioid peptides have also been found to act intracellularly, activating specific nuclear receptors and signaling through a modality that has been referred to as at the “intracrine” patterning, playing a major role in stem cell cardiogenesis [7,8,9].

The aim of this Special Issue that contains seven Research Articles, and three Review Articles, is to provide novel findings on the mechanistic bases underlying the cellular and molecular consequences of endorphinergic modulation, considering both the agonist- and receptor-related sites of signaling, their short- and long-lived intracellular patterning, and the future perspectives emerging at the basic and translational/clinical levels.

The Original Articles address a wide range of issues, including: (i) the analysis of the gene expression of anti-stress opioid-like players and of endogenous opioid receptor agonists under experimental model conditions exploring the complexity of the innate hypersensitivity to stress within the context of excessive ethanol consumption, or tobacco smoke/e-cigarette vapor; (ii) the dissection of opioidergic signaling in the modulation of energy balance, and interconnected metabolic patterning, including the interactions among opioid, adrenergic, and antioxidant systems; (iii) the investigation of opioidergic mechanisms in complex, and still largely unexplored, functions, such as the modulation of visual processing; (iv) the development and characterization of compounds harboring hybrid agonistic and antagonistic features that intriguingly unfold into both analgesic and anticancer properties; (v) the development of experimental models suitable for the study and the development of biased opioid receptor agonists, exhibiting remarkable analgesic but reduced side effects.

Regarding the first aspect (i), Caputi et al. [10] used genetically selected Marchigian Sardinian alcohol-preferring (msP) rats as an experimental model resembling a subset of *alcohol use disorder (AUD) patients* consuming excessive amounts of ethanol to experience self-relief from negative moods and the innate hypersensitivity to stress. Their results provided evidence for an innate upregulation of the corticotropin-releasing factor (CRF)-related system, together with an inherent upregulation of the opioid-like peptide nociceptin/orphanin FQ (N/OFQ), mediating negative mood and stress responses, at the level of both the amygdala (AMY) and the bed nucleus of the stria terminalis (BNST) of msP rats. The authors found that voluntary alcohol consumption dampened N/OFQ, as well as dynorphin, and CRF transmissions in the AMY, while eliciting more complex remodeling in the BNST of msP rats, by enhancing CRF and decreasing N/OFQ transcripts.

Remaining within the context of the first issue (i), Carboni et al. [11] investigated key neurotransmitter patterning in an experimental mouse model of nicotine addiction, another severe threat to public health. Withdrawal from intermittent cigarette smoke, or e-cigarette vapors, increased depressive and anxiety/obsessive–compulsive-like behaviors, also leading to cognitive impairment. Consonant with the take-home message from the study of Caputi et al. [10], an involvement of the CRF-related system was observed, in this case, with an increase in CRF and Crf1 mRNA levels, and a significant downregulation of nociceptin precursor expression upon smoke-withdrawal in the caudate-putamen nucleus (CPu). Smoke withdrawal also decreased brain-derived neurotrophic factor (BDNF) gene expression in the hippocampus, but enhanced its mRNA levels in the CPu. E-cigarette exposure also evoked an increase in delta opioid receptor transcripts in the CPu. On the whole, alterations in peptidergic signaling are suggested to exert a causative role in hampering hippocampal and striatal neuroplasticity following long-term smoke withdrawal.

Regarding the second aspect (ii), Seoane-Collazo et al. [12] provided evidence for a remarkable involvement of opioidergic signaling in nicotine-induced negative energy balance and body weight loss. The expression of the dynorphin precursor, as well as of the κ opioid receptor and p70 S6 kinase/ribosomal protein S6 signaling, was inhibited in the lateral hypothalamic area (LHA), following nicotine treatment. Such inhibitory effects were consistently mitigated in κ opioid receptor-deficient mice, and after κ opioid receptor antagonism, or specific knockdown at the LHA level.

A further implication of opioidergic signaling in the modulation of complex metabolic fate (ii) has been provided by Root-Bernstein et al. [13] who explored the functional interactions among opioids and amines, their receptors, and glutathione, with particular emphasis on the relative binding affinities of ascorbic acid, dehydroascorbic acid, and opioid and adrenergic compounds, and on glutathione and glutathione-like peptides derived from the extracellular loop regions of the human beta 2-adrenergic, dopamine D1, histamine H1, and μ opioid receptors. Their observations that some cysteine-containing peptides derived from these receptors bind ascorbic acid and/or dehydroascorbic acid, and that the same peptides generally bind opioid compounds, considered together with the evidence that glutathione not only binds morphine but even naloxone, methadone, and methionine enkephalin, corroborate novel bases for deciphering intertwined metabolic fates emerging from the interaction of opioidergic, adrenergic, and antioxidant systems. As suggested by the authors, this interactome may be operative during anesthesia and/or drug abuse, prompting further dissection of potential regulatory mechanisms.

Concerning the third issue (iii), the presence of β-endorphin-preferring, μ-opioid receptors (MORs) within the melanopsin-expressing intrinsically photosensitive retinal ganglion cells (ipRGCs) has stimulated the interest of Cleymaet et al. [14] in investigating the potential role of an MOR system in ipRGCs. The authors found that the MOR-selective agonist [D-Ala^2^, MePhe^4^, Gly-ol^5^]-enkephalin (DAMGO) abrogated the pupillary light reflex (PLR) evoked by light with intensities below the melanopsin activation threshold, but did not affect the PLR evoked through the activation of melanopsin signaling by bright blue irradiance. A selective MOR antagonist, or genetic ablation of MORs in ipRGCs, slightly enhanced dim-light-evoked PLR, but not that evoked by a bright blue stimulus. These data provide evidence that an opioidergic system is deeply involved in the modulation of non-image-forming visual processes.

The above-reported findings highlight the relevance of opioidergic signaling in the fine-tuning of cellular dynamics, and they prompt the need for developing novel synthetic compounds (iv) capable of further refining/expanding the repertoire of action exhibited by naturally occurring opioid peptides. To this end, Matalińska et al. [15] exploited the features of a hybrid compound (AA3266) that had been designed to encompass the traits of an opioid receptor agonist and a neurokinin-1 receptor (NK1R) antagonist, plus a selective cytotoxic action. In vivo, the authors observed a potent antinociceptive activity in an acute pain model. Upon a prolonged administration, the hybrid compound exhibited an analgesic effect comparable to, but with less tolerance than, that elicited by morphine. Moreover, unlike morphine, AA3266 did not cause constipation, a major side-effect in opioid use.

Intriguingly, the same hybrid exerted a strong cytotoxic action toward cancer cell lines while damaging noncancer normal cells at a significantly smaller extent.

In silico methods were also deployed to investigate the interactions of AA3266 with MOR and NK1R.

Consonant with the attempt of developing novel compounds with enhanced potency and properties, but with lesser side-effects (v), has been the in vitro analysis of biased μ opioid receptor (MOR) agonists in the study performed by Zhang et al. [16]. Although nonhuman primates (NHPs) represent an excellent translational model to assess the behavioral effects of candidate medications, the lack of studies on the biased signaling of agonists at the level of NHP MOR induced the authors to characterize MOR ligand bias in rhesus macaques. For this purpose, they used agonists that had been previously shown to exhibit different signaling bias at rodent and human MORs. With this strategy, the authors could provide evidence for a high degree of concordance between rhesus macaque and human MOR receptor signaling bias for all the agonists tested, thus paving the way for future translational behavioral studies based on the same approach.

These articles present several comprehensive up-to-date information on the modulatory actions of opioidergic systems, and they provide an in-depth analysis of the complexity of downstream signaling and functional implication of targeted spliced variants. Within this context, Brejchova et al. [17] discussed the expression of opioid receptors and the action of opioid drugs in cells of the immune system in animal models, as well as in humans. The authors analyzed some controversial issues on this topic, and highlighted how the analysis of the expression levels of opioid receptors in human peripheral blood lymphocytes may help to evaluate the outcome of methadone maintenance therapy in former opioid addicts, or could serve as a biomarker for chronic pain diagnosis.

The review article by Abrimian et al. [18] provided a detailed analysis of the extensive alternative pre-mRNA splicing of the μ opioid receptor (MOR) gene, OPRM1, focusing on the full-length seven (7)-transmembrane (TM) carboxyl (*C*)-terminal variants. The authors summarized the results from early pharmacological studies on the responses elicited by several endogenous opioid peptides through the mouse, rat, and human OPRM1 7TM C-terminal variants, underlying the relevance of further exploring opioidergic signaling in light of the novel mechanistic insights provided by this emerging field of enquiry.

Finally, Berthézène et al. [19] addressed the intriguing issue of the role played by opioids in tissue regeneration. They focused their discussion on the complex dynamics occurring in damaged tissues, where the local patterning may progress up to the level of scar healing, highlighting the substantial difference (s) between this type of rescue and the true tissue regeneration. In considering the latter process as a healing path regaining all the structural and functional attributes of the original tissue, the authors pointed at the major role exerted by nociceptive stimuli and signaling in providing an early pro-inflammatory milieu that may oppose the onset of scar healing. To this end, a significant amount of data were reviewed, suggesting that opioids (both endogenous and exogenous), by opposing an initial pro-inflammatory phase, while silencing nociceptive signaling, may concomitantly contribute to hamper effective tissue regeneration, with the final outcome of tissue remodeling and scarring.
